# Supplementary material for: Cell division orientation is coupled to cell–cell adhesion by the E-cadherin/LGN complex
Source: Nat Commun. 2017 Jan 3;8:13996. doi: 10.1038/ncomms13996 (PMC5216124; doi:10.1038/ncomms13996)
Supplement: Supplementary Information — Supplementary Figures and Supplementary References [file ncomms13996-s1.pdf]

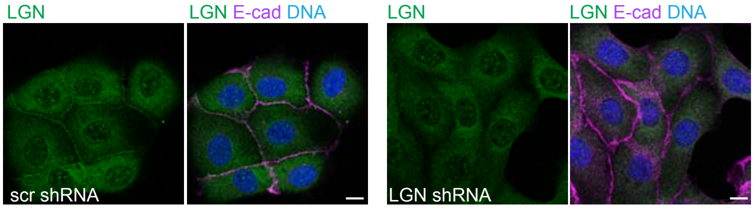

Supplementary Fig. 1. LGN staining after LGN shRNA expression
Level of LGN fluorescent staining in scr shRNA infected control MDCKs, or after shRNA mediated depletion of LGN. E-cadherin (E-cad) marks the cell-cell contacts. Scale bar, 10 μm.

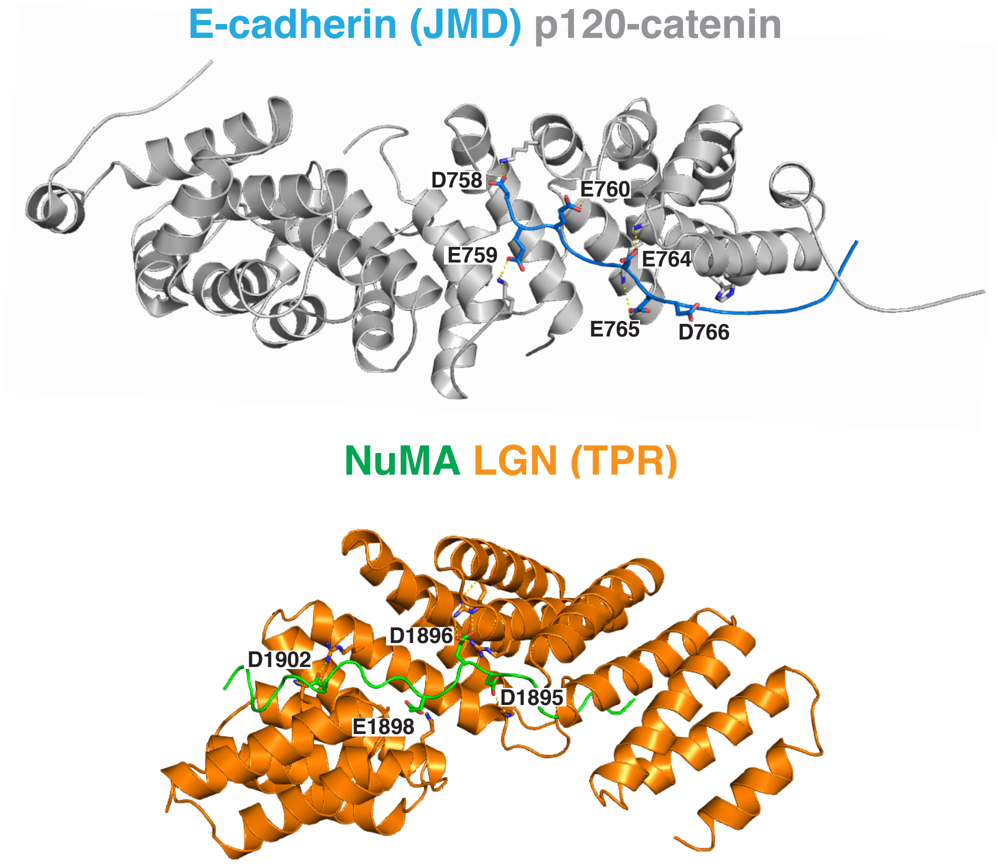

Supplementary Fig. 2. Structures of the p120/E-cadherin and LGN/NuMA complex
Three-dimensional structure of NuMA in complex with the TPR repeats of LGN [1] and of p120-catenin with E-cadherin [2], showing the negatively charged residues within NuMA and E-cadherin that establish binding to LGN and E-cadherin, respectively.

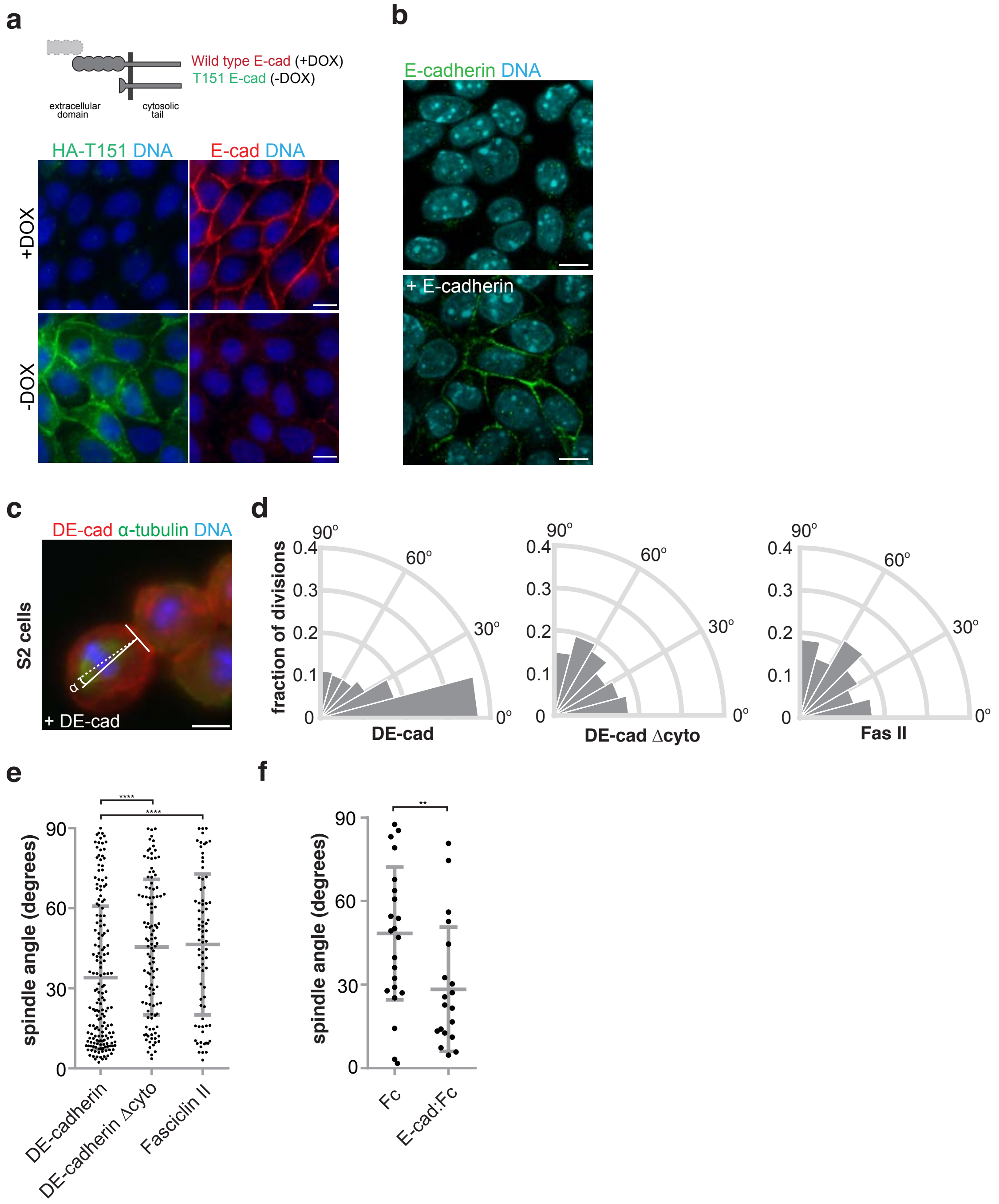

Supplementary Fig. 3. E-cadherin is an instructive cue for spindle orientation

(a) Schematic representations of full-length and T151 truncated E-cadherin under control of the doxycycline (DOX) repressible promotor, and immunostaining of endogenous E-cadherin (red) and HA-tagged T151 E-cadherin mutant (green) in the presence (+DOX) or absence (-DOX) of doxycycline, showing loss of endogenous E-cadherin but maintenance of a cohesive cell monolayer upon T151 expression (-DOX).

(b) Staining of E-cadherin in L cells, upon dexamethasone-induced expression of exogenous E-cadherin or an empty vector control.

(c) α-Tubulin staining of the mitotic spindle in Drosophila S2 cells stably expressing DE-cadherin.

(d) Rose diagrams of the mitotic spindle angle relative to the cell-cell contact in S2 cells stably expressing either DE-cadherin (DE-cad; n = 172), DE-cadΔcyto (n = 114), or Fasciclin II (Fas II; n = 76), binned from 3 independent experiments.

(e) Unbinned data of the mitotic spindle angle relative to the cell-cell contact in Drosophila S2 cells, shown as rose diagrams of binned data in Supplementary Fig. 3d.

(f) Unbinned data of the mitotic spindle angle relative to the cell-sidewall contact in MDCKs cells associated with E-cad:Fc or Fc control sidewalls, which is shown as rose diagrams of binned data in Fig. 3e.

Scale bars represent 10 μm. **** $p < 0.0001$; ** $p = 0.005$.

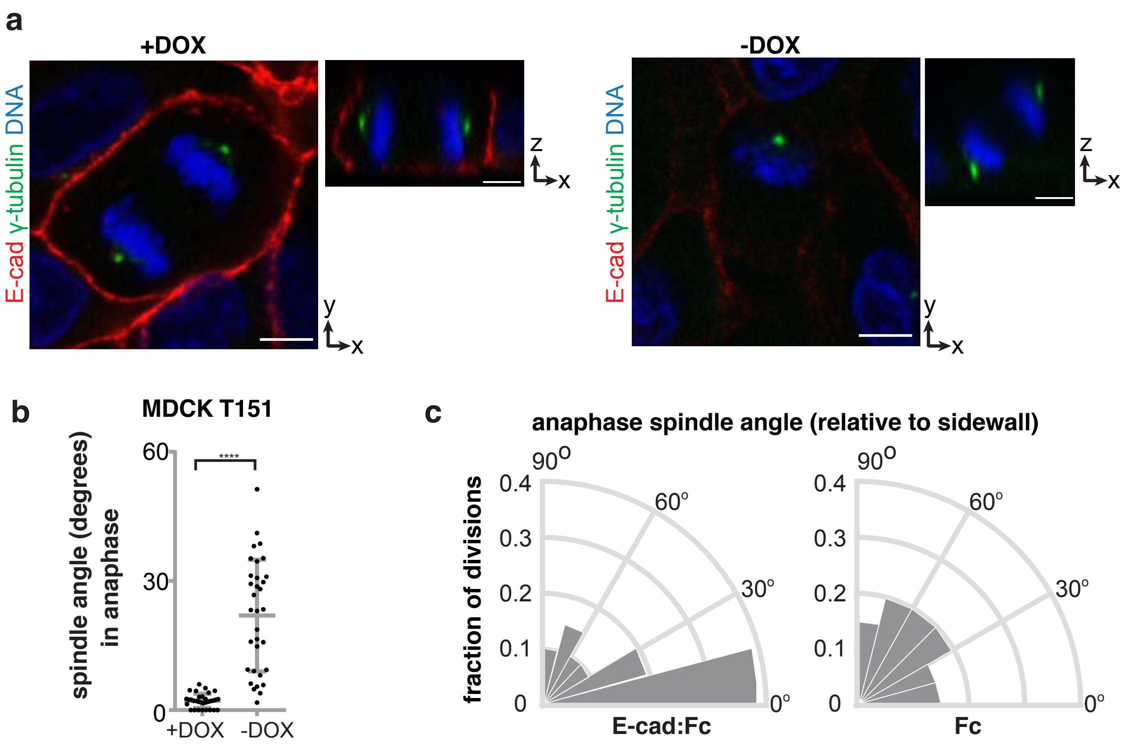

Supplementary Fig. 4. E-cadherin is an instructive cue for mitotic spindle orientation in anaphase.
(a) Confocal images of γ-tubulin labeled centrosomes in MDCK T151 cells showing the orientation of the mitotic spindle in anaphase cells.
(b) Quantification of mitotic spindle angle relative to the basal surface in MDCK T151 cells in anaphase.
(c) Rose diagram quantification of the mitotic spindle angle in anaphase in GFP-tubulin expressing MDCK cells associated with sidewalls coated with either E-cadherin:Fc ($n = 20$) or Fc ($n = 20$) control in anaphase cells.
Scale bars represent 5 μm. **** $p < 0.0001$.

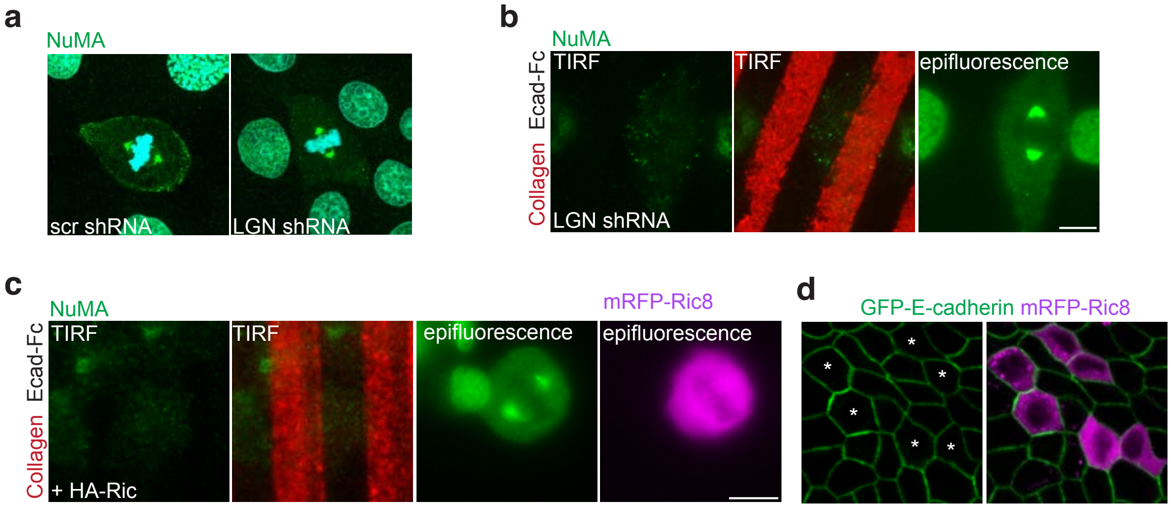

Supplementary Fig. 5. LGN/Gαi-dependent recruitment of NuMA to E-cadherin adhesions.
(a, b) shRNA-mediated depletion of LGN in MDCK cells, resulting in loss of NuMA from mitotic cell-cell contacts as shown by a Z-projection of confocal images of NuMA in MDCK monolayers (a), and by TIRF microscopy of NuMA in cells plated on micro-patterned alternating stripes of collagen-IV/E-cad:Fc (b). Quantification of data is shown in Fig. 5g.
(c) TIRF microscopy of NuMA in MDCK cell over-expressing mRFP-Ric8 to disrupt LGN/Gαi interactions, plated on micro-patterned alternating stripes of collagen-IV/E-cad:Fc. Quantification of data is shown in Fig. 5g.
(d) Localization of GFP-tagged E-cadherin in MDCK cells in the absence or presence or mRFP-Ric8 expression, showing the localization of E-cadherin at cell-cell contacts is unaffected in cells with Ric8 over-expression (marked with asterisks). Scale bars represent 10 μm.

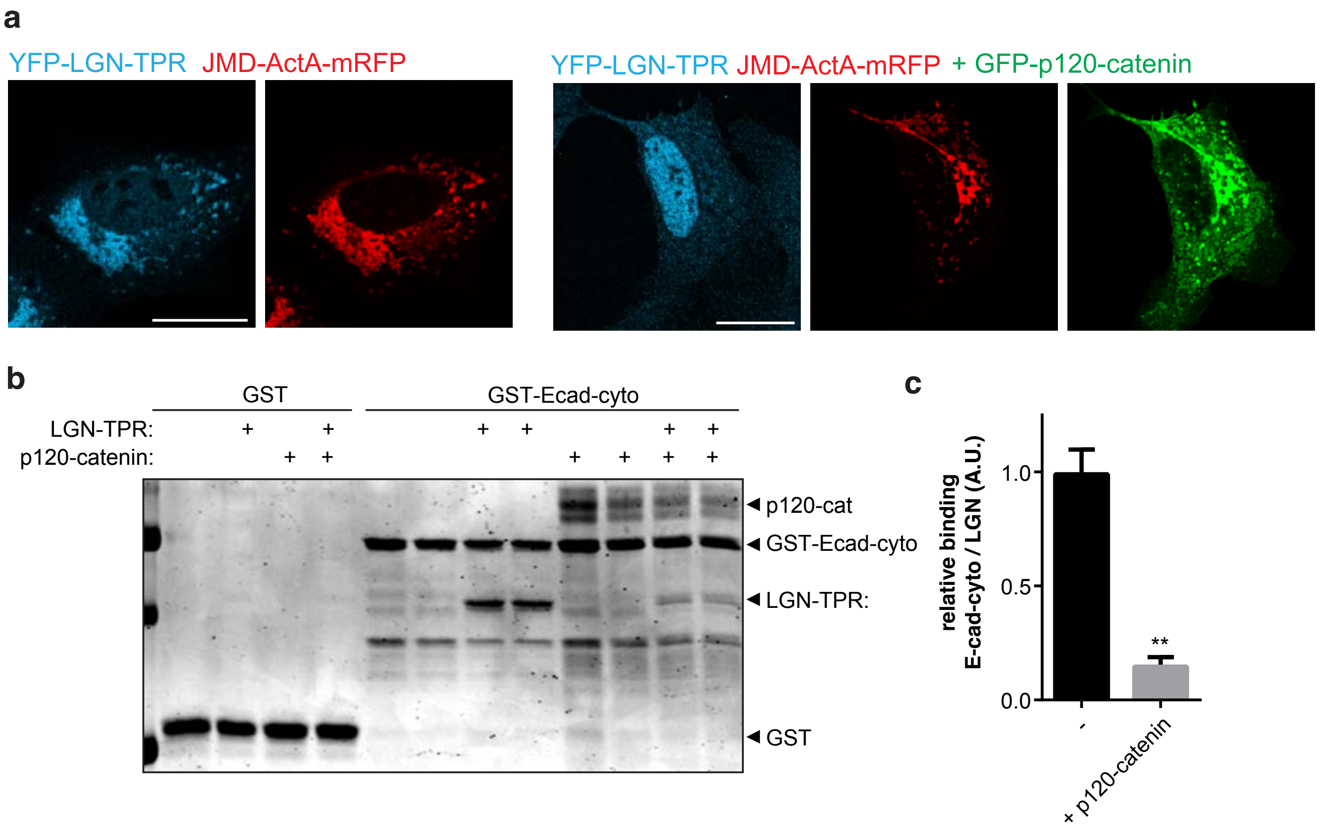

Supplementary Fig. 6. Binding of LGN and p120-catenin to E-cadherin JMD is mutually exclusive

(a) Recruitment of YFP-tagged LGN-TPR to E-cadherin Juxtamembrane domain (JMD) that was targeted to mitochondria by fusion with the mitochondrial localization signal (MLS) of ActA, which was lost upon co-expression of GFP-tagged p120-catenin.

(b) GST-pull down of recombinant E-cadherin cytosolic tail (GST-E-cad cyto) with LGN-TPR, in the absence or presence of recombinant p120-catenin.

(c) Quantification of the binding of recombinant LGN-TPR to E-cadherin cytosolic tail, in the absence or presence of recombinant p120-catenin, with average and SD from three independent experiments. Together, these data demonstrate mutual exclusive binding of p120-catenin and LGN to E-cadherin. Of note, p120-catenin binding increases the life-time of E-cadherin at the plasma membrane by sterically hindering accessibility of an endocytic motif adjacent to the p120-catenin (and LGN) binding site (ref. 3), and the comparable modes of interaction suggest that LGN binding may also affect E-cadherin internalization.

Scale bars represent 10 μm. ** $p < 0.003$.

Supplementary References

1. Zhu, J. et al. LGN/mInsc and LGN/NuMA complex structures suggest distinct functions in asymmetric cell division for the Par3/mInsc/LGN and Galphai/LGN/NuMA pathways. Mol. Cell **43,** 418–431 (2011).
2. Ishiyama, N. et al. Dynamic and Static Interactions between p120 Catenin and E-Cadherin Regulate the Stability of Cell-Cell Adhesion. Cell 141, 117-128, (2010).
3. Reynolds, A. B. p120-catenin: Past and present. Biochim Biophys Acta 1773, 2-7, (2007)
